# P-472. Evaluating and Refining the Candida Score for Invasive Candidiasis in Critically Ill Non-Neutropenic Children

**DOI:** 10.1093/ofid/ofaf695.687

**Published:** 2026-01-11

**Authors:** Puttichart Khantee, Kochakorn Pinichkijpaisal, Kamolwish Laoprasopwattana

**Affiliations:** Faculty of Medicine, Prince of Songkla University, Hatyai, Songkhla, Thailand; Faculty of Medicine, Prince of Songkla University, Hatyai, Songkhla, Thailand; Prince of Songkla University, Songkla, Pattani, Thailand

## Abstract

**Background:**

Delayed antifungal treatment of invasive candidiasis (IC) in critically ill children is associated with increased morbidity and mortality. While the Candida score is widely used in critically ill non-neutropenic adults, its applicability in pediatric patients remains unvalidated. This study aimed to assess the performance of the original Candida score in critically ill non-neutropenic pediatric patients and to develop and internally validate a Pediatric Candida score.

**Table 1.**
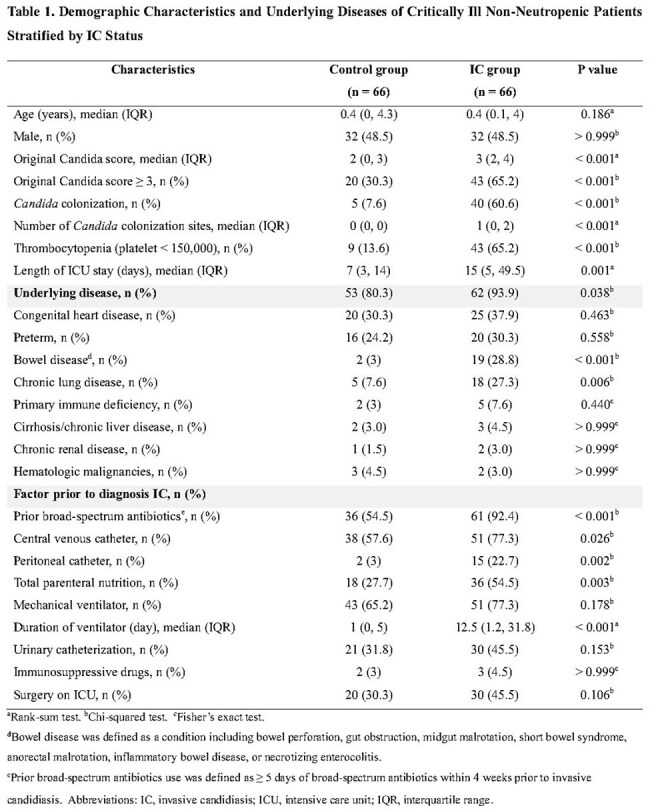
Demographic Characteristics and Underlying Diseases of Critically Ill Non-Neutropenic Patients Stratified by Invasive candidiasis Status

**Table 2.**
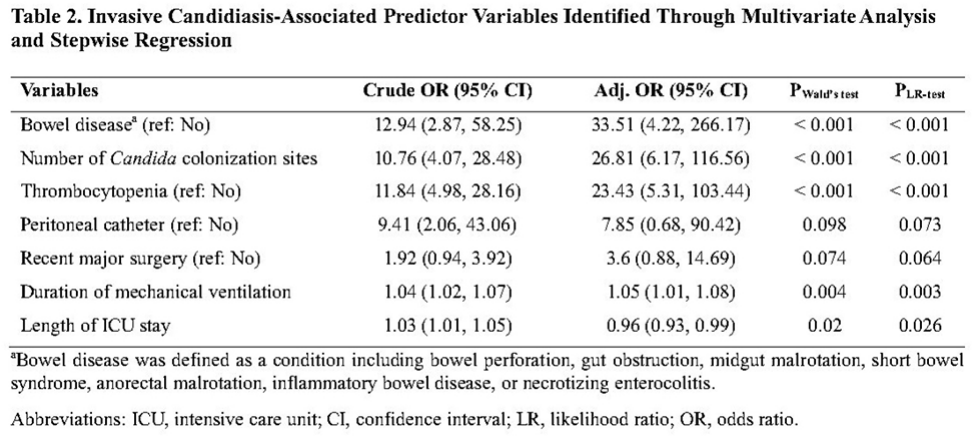
Invasive Candidiasis-Associated Predictor Variables Identified Through Multivariate Analysis and Stepwise Regression

**Methods:**

A retrospective matched case-control study was conducted at Songklanagarind Hospital, Thailand (2009–2023). Sixty-six critically ill non-neutropenic children with proven IC were matched by age, sex, and time at risk with 66 controls. Risk factors were identified using univariate analysis and parsimonious logistic regression to formulate the Pediatric Candida score.

**Table 3.**
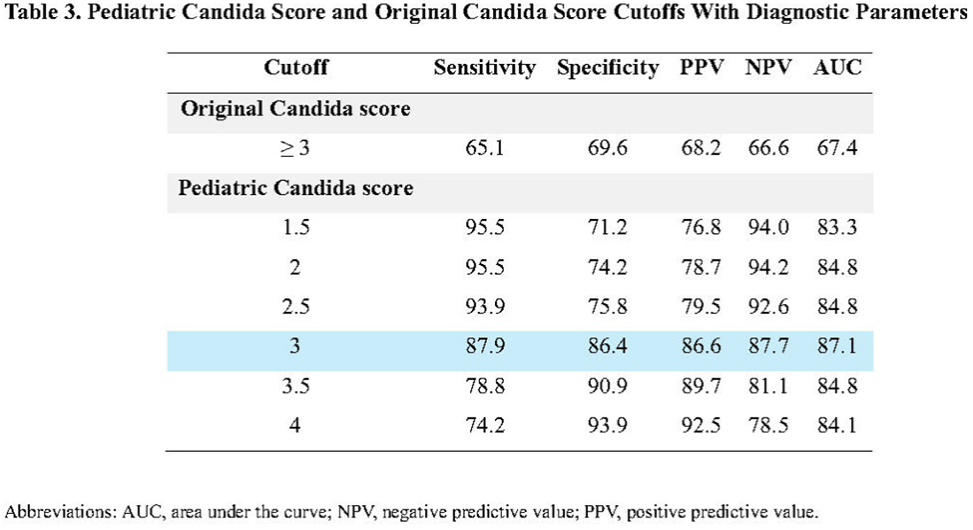
Pediatric Candida Score and Original Candida Score Cutoffs With Diagnostic Parameters

**Figure 1.**
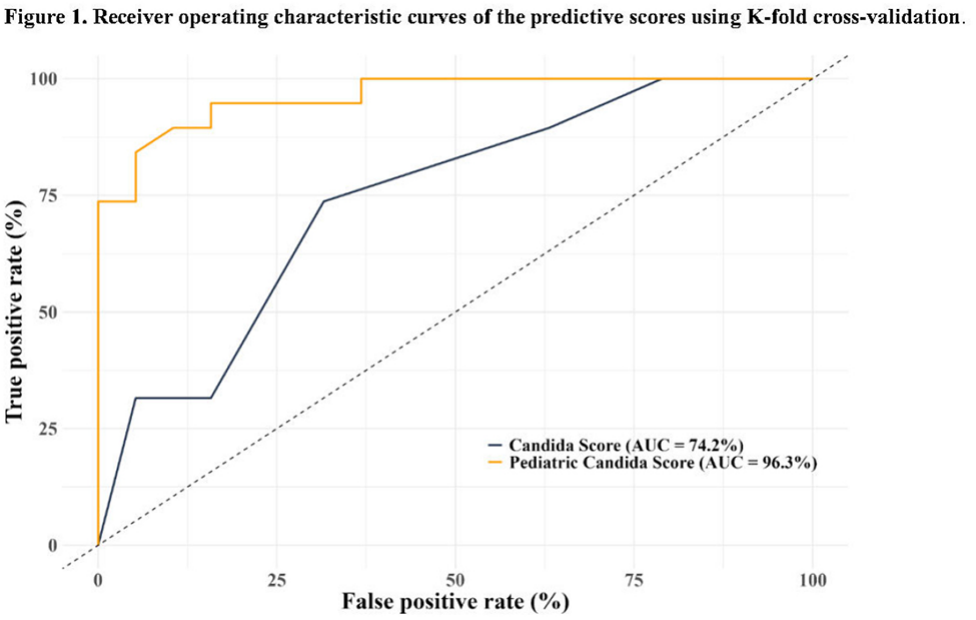
Receiver operating characteristic curves of the predictive scores using K-fold cross-validation.

**Results:**

From the parsimonious model, the Pediatric Candida score included the following risk factors: bowel disease (odds ratio [OR], 33.51; 95% confidence interval [CI]: 4.22–266.17, P< 0.001), number of *Candida* colonization sites (OR: 26.81; 95%CI: 6.17–116.56, P< 0.001), thrombocytopenia (OR: 23.43; 95%CI: 5.31–103.44, P< 0.001), duration of mechanical ventilation (OR: 1.05; 95%CI: 1.01–1.08, P = 0.003), length of ICU stay (OR: 0.96; 95%CI: 0.93–0.99, P = 0.026), peritoneal catheter use (OR: 7.85; 95%CI: 0.68–90.42, P = 0.073), and prior surgery (OR: 3.6; 95% CI: 0.88–14.69, P = 0.064). The formula:

Pediatric Candida Score = 3.29×(Colonization sites) + 3.15×(Thrombocytopenia) – 0.04×(ICU days) + 3.51×(Bowel disease) + 2.06×(Peritoneal catheter) + 0.04×(Ventilation days) + 1.28×(Surgery). Compared to the original Candida score (sensitivity 65.1%, specificity 69.6%), the Pediatric Candida score showed superior performance (sensitivity 87.9%, specificity 86.4%, accuracy 87.1%).

**Conclusion:**

The Pediatric Candida score outperformed the original Candida score, demonstrating superior diagnostic accuracy for IC in critically ill, non-neutropenic children. This tailored tool supports early diagnosis and timely antifungal therapy, potentially improving outcomes in vulnerable populations. Future multicenter validation is necessary to confirm its clinical utility and optimize pediatric antifungal stewardship.

**Disclosures:**

All Authors: No reported disclosures

